# Effect of maturation time and adhesive system on the shear bond strength and failure modes of Biodentine™ bonded with resin composite and glass ionomer cement

**DOI:** 10.2340/biid.v13.45898

**Published:** 2026-04-27

**Authors:** Wichida Chaweewannakorn, Kittichot Saetae, Achirawat Wattakul, Suprawee Ngamjantratip, Pathida Pornraksamanee, Nirada Dhanesuan, Narinee Chinajitphan, Kwanchanok Youcharoen

**Affiliations:** a Department of Pedodontics and Preventive Dentistry, Faculty of Dentistry, Srinakharinwirot University, Bangkok, Thailand; b Faculty of Dentistry, Srinakharinwirot University, Bangkok, Thailand; c Department of Stomatology, Faculty of Dentistry, Srinakharinwirot University, Bangkok, Thailand

**Keywords:** Biodentine™, failure mode, high-viscosity glass ionomer cement, resin composite, shear bond strength

## Abstract

**Objectives:**

This study aimed (1) to compare the shear bond strength (SBS) and failure modes of Biodentine™ (BD) specimens bonded with resin composite (RC) or high-viscosity glass ionomer cement (HVGIC) at different maturation times and (2) to compare the SBS of BD bonded with RC using different adhesive systems.

**Materials and Methods:**

Part I: A total of 120 BD specimens were bonded with either RC or HVGIC (*n* = 60 each), then divided into four groups (*n* = 15 each) based on BD maturation time: 12 min, 24 h, 48 h, and 1 week. Part 2: A total of 45 BD specimens matured for 24 h were bonded with RC using 3 adhesives (*n* = 15 each): (1) Single Bond Universal (SU) with self-etched technique (SU-SE), (2) Adper Single Bond 2 adhesive with etch and rinse technique (AS-ER), and (3) SU adhesive with etch-and-rinse technique (SU-ER). All specimens underwent pH cycling and thermocycling. SBS was measured using a universal testing machine, and failure modes were examined under a stereomicroscope.

**Results:**

BD bonded with RC showed significantly higher SBS than BD bonded with HVGIC at all BD maturation times (*p* < 0.05). No significant SBS differences were found within RC groups, except between the 24-h and 1-week. Cohesive failure predominated in RC specimens (70%), whereas adhesive failure prevailed in HVGIC specimens (71.7%). SU-ER had significantly higher SBS than SU-SE or AS-ER (*p* < 0.05).

**Conclusion:**

BD bonded with RC demonstrated higher SBS than BD bonded with HVGIC. The failure mode in the RC group was predominantly cohesive, whereas in the HVGIC group, it was primarily adhesive. These findings support the clinical applicability of immediate definitive RC placement over BD using a methacryloyloxydecyl dihydrogen phosphate-containing adhesive with an etch-and-rinse strategy.

KEY MESSAGES

- The recommended 12-min setting time for Biodentine™ appears adequate, as delayed restoration had minimal influence on bonding performance with resin composite.
- This study supports immediate definitive resin composite placement over Biodentine™ using a methacryloyloxydecyl dihydrogen phosphate (MDP)-containing adhesive with an etch-and-rinse strategy.

## Introduction

Dental caries remains the most prevalent oral health problem worldwide and affects over 2.5 billion people [1]. Modern approaches to managing dental caries have shifted from ‘extension for prevention’ to minimally invasive dentistry, emphasizing conservative strategies to preserve natural tooth structure and pulp vitality. Advancements in adhesive and bioactive dental materials, which promote a peripheral seal and facilitate caries arrest, have rendered extensive removal of tooth structure and complete caries excavation unnecessary [2, 3].

The introduction of hydraulic calcium silicate-based cements (CSCs) such as Mineral Trioxide Aggregate (MTA), Biodentine™ (BD), Bioaggregate, and TheraCal has attracted attention as alternative pulp capping materials for deep caries management due to their good sealing properties, biocompatibility, marginal adaptation, and ability to induce dentine bridge formation with minimum pulp inflammation [4–6]. BD, developed by Septodont in Saint-Maur-des-Fossés Cedex, France, is a tricalcium silicate-based cement introduced in 2009 as a bioactive dentine substitute. BD and MTA have demonstrated comparable biological behavior in both primary and permanent teeth [7, 8]. However, BD offers advantages in handling characteristics, shorter setting time, and improved aesthetic outcomes. In addition, BD exhibits favorable mechanical properties, including higher compressive strength, surface hardness, flexural strength, and elastic modulus than other calcium silicate cements, including MTA [9]. Consequently, BD has been widely applied clinically as a root-end filling agent, apexification material, pulp capping agent, permanent dentine substitute, and as a reparative material for perforations [10]. BD is contained in a capsulated powder and a pipette containing liquid for use in a triturator, making it user-friendly. The setting time of BD, as reported by the manufacturer, is 9–12 min [11]. However, other studies have indicated that the material may require between 48 min and up to 2 weeks to achieve complete setting [12, 13]. This discrepancy raises an important clinical consideration regarding the optimal timing for final restoration placement, whether it should be performed immediately or delayed to ensure material stability and integrity.

The placement of a well-adapted permanent restoration, coupled with the optimal bond between the restorative material, tooth substrate, and pulp capping agent, is vital for the clinical success of pulp capping procedures as it prevents bacterial microleakage and ensures long-term treatment outcomes [14]. Shear bond strength (SBS) is a critical parameter that reflects the strength of the bond at the interface between the biomaterial and restorative material. As masticatory forces predominantly involve shear stress, SBS serves as a reliable indicator of the material’s adhesive behavior under functional conditions. Moreover, establishing a durable SBS between the bioactive material and the restorative component is essential for limiting bacterial microleakage and ensuring optimal clinical outcomes in vital pulp therapy [15]. Additionally, the quality and effectiveness of the bonding can be assessed by analyzing the failure modes categorized as cohesive, adhesive, or mixed [16, 17].

Various restorative materials can be applied over BD. Resin composite (RC) and glass ionomer cements (GICs) are particularly popular in restorative dentistry for their superior aesthetic qualities. GIC can be considered as a basic restorative material due to its ability to chemically bond to dentine, release fluoride, and its ease of application, which allows for bulk placement without the need for adhesives [18, 19]. RC is extensively employed as a restorative material in contemporary dentistry; however, when applied over BD, the adhesive bond strength between BD and RC is a critical determinant of clinical success. According to studies on resin adhesion strategies, adhesives can be categorized into etch-and-rinse (ER) and self-etch (SE) systems. While the ER systems use phosphoric acid to remove the smear layer, the SE systems use acidic monomers instead. Growing clinical interest in simplified and less technique-sensitive adhesive systems has led to the development of a new class of adhesives known as universal adhesives. These adhesives can be applied using both ER and SE techniques and are capable of bonding to a broad range of substrates, including metals, zirconia ceramics, and dental hard tissues [20]. This variety of available restorative materials and adhesive systems necessitates a critical evaluation of their compatibility with BD, with particular emphasis on optimizing bonding performance. Furthermore, the majority of studies have primarily investigated the bond strength of BD in static aging conditions. However, it is crucial to assess the bond strength of this material in environments that closely mimic the conditions of the human oral cavity. The most commonly employed methods for aging dental materials are pH cycling and thermocycling. Therefore, this study aimed to (1) compare the SBS and failure modes of BD bonded with RC and high-viscosity glass ionomer cement (HVGIC) at different maturation times of BD and (2) evaluate the SBS between BD and RC using different adhesive systems. The tested null hypothesis was that there would be no statistically significant difference in the bond strength between the restorative material and BD at different maturation times. Secondly, the type of adhesive material would have no effect on the bond strength between BD and RC.

## Material and methods

### BD specimen preparation

#### Part 1

**Figure 1:**
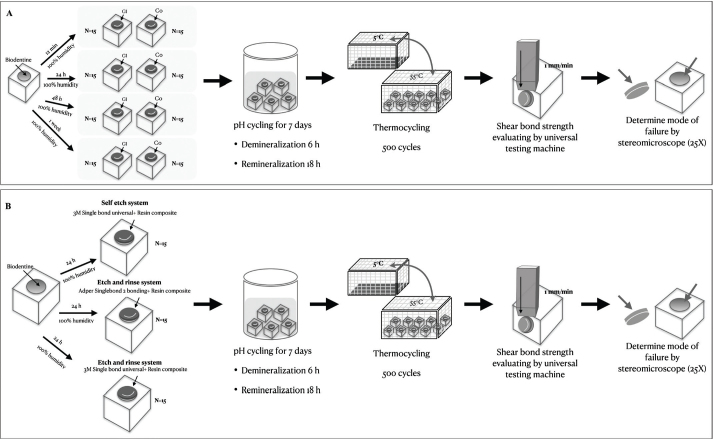
Schematic diagram of the procedures used for evaluating shear bond strength and mode of failure between Biodentine™ and resin composite or high-viscosity glass ionomer cement.

A total of 120 BD specimens were prepared in prefabricated acrylic molds containing a cavity with an 8-mm diameter and a 2-mm height. BD (Septodont, Saint-Maur-des-Fossés, France) was mixed according to the manufacturer’s instructions and applied to the cavities. A glass slab was placed on top of the mold to create standardized surfaces. All 120 specimens were randomly divided into two groups based on restorative material: RC with Single Bond Universal (SU) adhesive with etch-and-rinse technique (SU-ER) (*n* = 60) or HVGIC (*n* = 60). For each material, the specimens were divided into four subgroups (*n* = 15 per group) according to BD maturation time: 12 min, 24 h, 48 h, and 1 week. The specimens were then kept in 100% humid environment according to the maturation time before placement of restorative material (Figure 1A).

#### Part 2

A total of 45 BD specimens were prepared following the same procedures as in part I. After 24 h maturation time in 100% humid environment, the specimens were randomly allocated to three adhesive groups (*n* = 15 per group) for bonding with RC. The three groups were: (1) SU with self-etched technique (SU-SE), (2) Adper Single Bond 2 adhesive with etch and rinse technique (AS-ER), and (3) SU-ER (Figure 1B).

The materials used, including chemical composition and instructions for use, are described in Table 1.

**Table 1:** Materials used in this study.

Material	Manufacturer	Composition	Application mode
Biodentine™ Lot no: B32340	Septodont, Saint-Maur-des-Fossés, France	Powder: Tricalcium silicate, dicalcium silicate, calcium carbonate and oxide, zirconium oxide, and iron oxide Liquid: calcium chloride, hydrosoluble polymer, water	1. Gently tap the BD capsule to loosen the powder. 2. Pour 5 drops of liquid into the capsule. 3. Place the capsule in a mixing device and mix for 30s at a high speed of 4,000 rotations per minute.
3M™ Single Bond Universal	3M, Deutschland GmbH, Neuss, Germany	10-MDP monomer, dimethacrylate resins, HEMA, Vitrebond copolymer, fillers, ethanol, water, initiators, silane	Etch-and- rinse strategy 1. Apply 37% phosphoric acid for f20s. 2. Rinse with water and dry with mild air flow. 3. Apply bond and mild air for 5s. 4. Light cure for 10s (3M ESPE Elipar Deep Cure-S LED, Seefeld, Germany). Self-etch strategy 1. Apply bond and mild air for 5s. 2. Light cure for 10s (3M ESEP Elipar Deep Cure-S LED, Seefeld, Germany).
3M™ Adper™ Single Bond 2 Adhesive	3M ESPE, St. Paul, MN, USA	Dimethacrylates resins, 3M™ Vitrebond™ copolymer, HEMA, water, ethanol, filler, and initiators	1. Apply 37% phosphoric acid for 20s. 2. Rinse with water and dry with mild air flow. 3. Apply bond and mild air for 5s. 4. Light cure for 10s (3M ESEP Elipar Deep Cure-S LED, Seefeld, Germany).
ESPE Filtek Z-350, 3M	3 M ESPE, St Paul, MN, USA	Bis-GMA, UDMA, Bis-EMA, TEGMA, PEGDMA, aggregated zirconia/silica cluster fillers (20 nm silica and 4–11 nm zirconia particles), nonagglomerated/ nonaggregated 20 nm silica filler, nonagglomerated/ nonaggregated 4–11 nm zirconia filler	Place RC in the mold and light cure for 40s (3M ESEP Elipar Deep Cure-S LED, Seefeld, Germany).
Fuji IX GP EXTRA	GC, Tokyo, Japan	Powder: Alumino-silicate glass, polyacrylic acid Liquid: Polyacrylic acid, water	1. Apply Dentine conditioner to the prepared cavity for 20s. Rinse thoroughly with water and gently dry the tooth surface without over-drying. 2. Activate the powder and liquid and mix for 10s with the amalgamator at high speed (4,000 rpm). 3. Load the capsule into the applicator gun and dispense the material directly into the mold and wait for 3 min setting time.
3M™ Scotchbond™ Universal Etchant	3 M ESPE, St Paul, MN, USA	Water, phosphoric acid, synthetic amorphous silica, fumed, crystalline- free, polyethylene glycol, aluminum oxide	
Dentine conditioner	GC, Tokyo, Japan	Distilled water, polyacrylic acid	

### pH cycling and thermocycling

To simulate the oral environment, the specimens were subjected to pH cycling for 14 days under cariogenic conditions using a demineralizing solution (1.5 mM CaCl_2_, 0.9 mM KH_2_PO_4_, 5 mM NaN_3_, 50 mM acetic acid at pH 5.0) for 6 h and then immersed in remineralizing solution (1.5 mM CaCl_2_, 0.9 mM KH_2_PO_4_, 130 mM KCl, 50 mM Hepes at pH 7.0) for 18 h [21]. After pH cycling, all specimens underwent thermocycling for 500 cycles between 5°C and 55°C (hot water bath [HWB332R], cold water bath [CWB332R], and temperature sensor [TC301]; King Mongkut’s Institute of Technology, Ladkrabang, Thailand) [22].

### Shear bond strength

After completion of the aging protocols, specimens were secured in a holder placed on the platform of the universal testing machine for SBS testing (EZ-LX, Shimadzu, Tokyo, Japan) at a crosshead speed of 1 mm/min. The SBS values were recorded in newton and converted to megapascals (MPa).

### Analysis of failure mode

**Figure 2:**
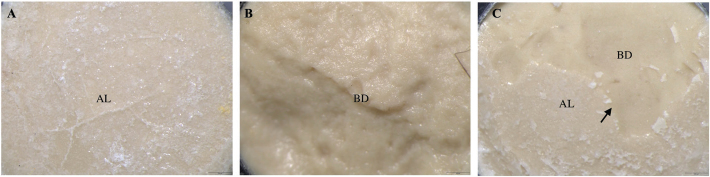
Representative stereomicroscopic images (×25 magnification; scale bar = 500 µm) illustrating the failure modes between Biodentine™ and restorative materials after the shear bond strength test. (BD: Biodentine™; AL: Adhesive layer). (A) Adhesive failure: Separation occurred at Biodentine™ and restorative material interface, with the adhesive layer visible at the interface. (B) Cohesive failure within Biodentine™. (C) Mixed failure: Combination of adhesive failure with adhesive layer at the interface and cohesive failure within Biodentine™. Arrow indicates the boundary between Biodentine™ and the adhesive layer.

After the SBS testing was performed, each debonded BD specimen was studied using a stereomicroscope (Olympus SZX7 and Olympus EP50, Olympus Corp., Tokyo, Japan) at 25X magnification by a single examiner, and the failure mode was classified into adhesive, cohesive, or mixed failures (Figure 2).

### Statistical analysis

Statistical analysis was performed using the SPSS software package version 20 (SPSS Inc., Chicago, IL, USA). Data were assessed for normality using the Shapiro–Wilk test. In Part 1, the SBS data in the RC and HVGIC groups were not normally distributed; therefore, the Kruskal–Wallis test was used to evaluate differences in SBS between groups, followed by post-hoc analysis using the Mann-Whitney U-test. In Part 2, normality was confirmed, and differences in SBS among the adhesive groups were analyzed using one-way analysis of variance (ANOVA) followed by Tukey’s Honestly Significant Difference (HSD) test. A significance level of *p* < 0.05 was used for all tests. Kappa statistics were calculated to assess intra-examiner reliability in the evaluation of failure modes.

## Results

The intra-examiner reliability for failure mode evaluation yielded a Kappa value of 0.85, indicating almost perfect agreement [23].

**Figure 3:**
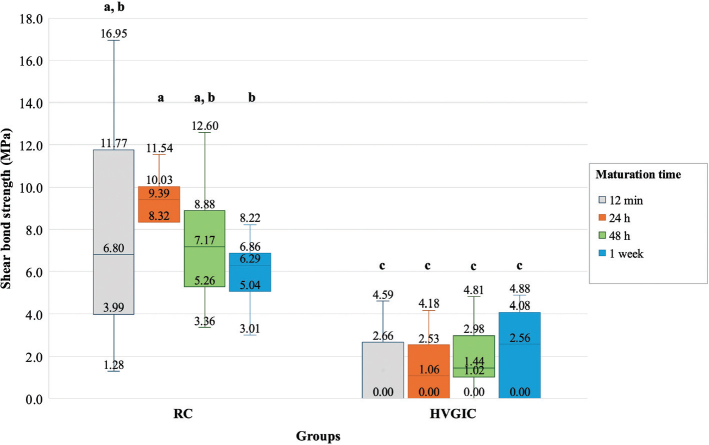
Comparison of shear bond strength of Biodentine™ bonded with resin composite or high-viscosity glass ionomer cement at different maturation times Different lowercase letters represent statistically significant differences between groups (*p* < 0.05). HVGIC: high-viscosity glass ionomer cement; RC: resin composite.

For the first part of the study comparing SBS at different maturation times, the median SBS was found to be highest in RC bonded with 24-h maturation time BD (median: 9.39; interquartile range [IQR]: 8.32, 10.03 MPa). The lowest SBS was found in HVGIC bonded with 12-min BD (median: 0; IQR: 0, 2.66 MPa). Overall, BD bonded with RC showed statistically significantly higher SBS than BD bonded with HVGIC in all maturation times tested (*p* < 0.05). Among the RC groups, there was no statistically significant difference across the four maturation times (*p* < 0.05), except between the 24-h and 1-week groups, where the 24-h group showed significantly higher SBS. In contrast, no statistically significant difference was observed among all maturation times among the HVGIC groups (Figure 3).

**Figure 4:**
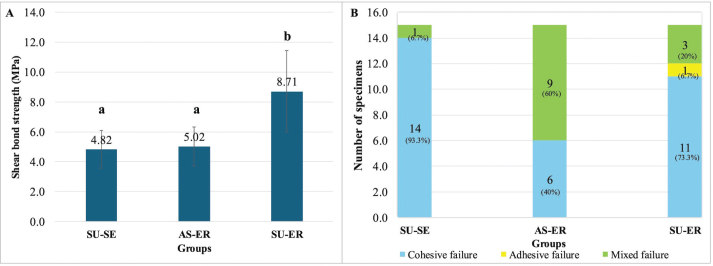
(A) Comparison of shear bond strength of Biodentine™ bonded with resin composite using different adhesive systems. Different lowercase letters represent statistically significant differences between groups (*p* < 0.05), (B) comparison of the failure mode of Biodentine™ bonded with resin composite using different adhesive systems. SU-ER: Single Bond Universal adhesive with etch-and-rinse technique; SU-SE: Single Bond Universal adhesive with self-etched technique; AS-ER: Adper Single Bond 2 Adhesive with etch-and-rinse technique.

**Figure 5:**
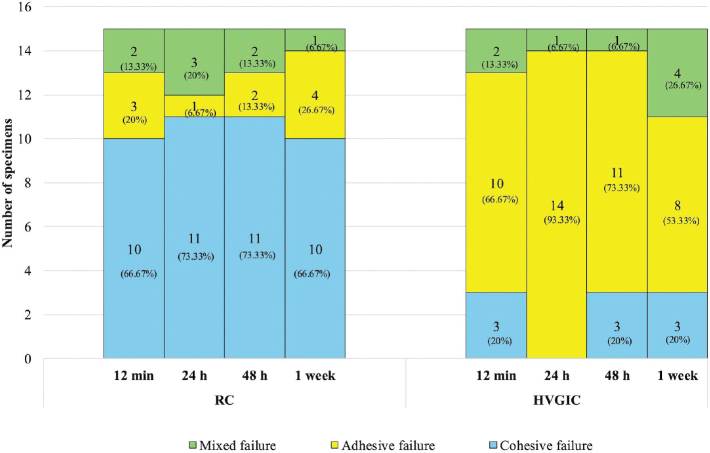
Comparison of failure mode of Biodentine™ bonded with resin composite or high- viscosity glass ionomer cement at different maturation times. HVGIC: high-viscosity glass ionomer cement; RC: resin composite.

Regarding the failure mode, most RC specimens (70%) exhibited cohesive failure, whereas adhesive failure predominated among HVGIC specimens (71.7%) (Figures 4B and 5).

For the second part of the study, when the SBS of different adhesive systems was investigated, the normality was confirmed by the Shapiro-Wilk test, and the difference in SBS between groups was analyzed using one-way ANOVA followed by *post hoc* comparisons. The highest SBS was observed in the SU-ER group (mean ± standard deviation [SD]: 8.71 ± 2.74 MPa), which was significantly higher than that of both the SU-SE (4.82 ± 1.27 MPa) and AS-ER (5.02 ± 1.31 MPa) groups (*p* < 0.05) (Figure 4A).

## Discussion

Calcium silicate cements are increasingly being used as dentine substitutes for pulp protection in the management of deep carious lesions due to their biocompatibility, biointeractivity, and bioactive properties [24–26]. The overall clinical success of caries management, apart from the materials used, is influenced by the quality and adequacy of their bond [27, 28]. As there are currently no specific guidelines regarding the restoration of teeth undergoing vital pulp therapy or deep caries management, this study was designed to investigate SBS between BD bonded with RC or HVGIC at different maturation times of the BD and the effect of adhesive systems on the bonding efficacy between BD and RC.

In this study, the SBS varied depending on the maturation time of BD and the type of restorative material used, leading to the rejection of the first null hypothesis. Thus, RC restored over BD exhibited significantly higher SBS than HVGIC at all BD maturation times, in accordance with previous studies [18, 29–31]. Celiksoz et al. reported that 12-min and 24-h BD bonded with RC demonstrated SBS of approximately 6 MPa, whereas EQUIA Forte HT, a GIC, showed an SBS ranging from 1.2 to 1.6 MPa [31]. These results are comparable to the findings of our study, although the GIC materials differed. Scanning electron microscope (SEM) analysis reported by Meraji and Camilleri [30] revealed a distinct wide gap at the interface between BD and GIC, indicating a weak bond between the two materials. This was further supported by the fact that all GIC specimens in their study were dislodged from BD during the demolding stage, reflecting very poor adhesion [30].

In the HVGIC groups, SBS remained low across all BD maturation times, with no statistically significant differences observed between the time points. This finding is consistent with a previous study that varied BD maturation times before bonding to HVGIC [31]. Based on the present results and prior research, the bonding mechanism between GIC and tricalcium silicate materials appears to be weaker than the reactions that occur between methacrylate monomers [18]. The inferior adhesion of HVGIC to BD may be attributed to several factors. During the early maturation phase of BD, GICs may absorb water from the calcium silicate matrix, thereby interfering with the hydration process of BD and potentially leading to incomplete hydration and increased interfacial porosity [31]. In addition, the bonding between BD and HVGIC may be limited by the absence of effective micromechanical interlocking and weaker chemical interaction at the interface [29]. This contrasts with resin-based systems, which benefit from both micromechanical retention and chemical bonding through functional monomer-calcium interactions. Furthermore, the setting reaction of CSCs generates an alkaline pH, whereas the setting reaction of GIC produces an acidic pH. This discrepancy may impair crystal formation and adversely affect the properties of calcium silicate–based materials, including strength, hardness, setting behavior, and solubility, potentially leading to microleakage. Thus, the low porosity of BD and the setting process may limit mineral deposition and crystal adhesion, thereby preventing effective micromechanical retention [32]. These findings suggest that layering GIC over BD, whether as a base or restorative material, may result in reduced bond strength. Therefore, direct restoration with RC is recommended over the use of GIC in such cases [29–31].

Another factor that may influence the bond strength between BD and restorative materials is the elapsed time since BD preparation, which is crucial for the material to achieve internal maturity. When considering RC bonded with SU-ER, delaying the placement of RC over BD to 1 week significantly reduced the SBS compared to placement at 24 h. However, no significant difference was found between 12-min, 48-h and 1-week groups. These results differ from previous studies [29, 33–35], which reported a significant reduction in SBS when RC was placed at an early maturation time (12 min) compared to a delayed placement (2 weeks) for BD. The discrepancies between the result of previous studies and the present findings may be attributed to differences in the adhesive systems and experimental protocols. In a study by Sismanoglu et al. [36], which applied Single Bond Universal as in our study, the bond strength between BD and RC was evaluated using the µSBS test across waiting times of 12 min, 24 h, and 1 week with various adhesive systems. When Single Bond Universal was used, the µSBS values showed no significant differences between immediate and delayed placement of RC, supporting the findings of the present study. Several other studies support the present findings, suggesting that early placement of RC over BD is recommended. Thus, Celiksoz et al. [31] reported that RC restorations placed over BD at 12 min exhibited similar SBS compared to those placed at 24 h. Similarly, Odabaş et al. [33] found no statistically significant difference in SBS between RC bonded with BD at 12 min and at 24 h. Palma et al. [37] extended the investigation to longer maturation times and reported comparable bonding efficiency between RC bonded with BD at 12 min and at 1 week. A common feature among these studies is the use of methacryloyloxydecyl dihydrogen phosphate (MDP)-containing adhesives, which may contribute to consistent bonding performance across different maturation times. The 10-MDP functional monomer enhances adhesion by chemically interacting with calcium ions released from BD, forming stable calcium-phosphate salts. Consequently, the presence of 10-MDP promotes additional chemical bonding that complements micromechanical retention, thereby improving the overall adhesive performance [29, 31]. These findings support the feasibility of immediate final restoration placement in a single visit. In the context of vital pulp therapy, completing the sealed restoration in a single visit may minimize the risk of leakage and enhance time efficiency [38].

However, several previous studies have reported higher bond strength with longer maturation times of calcium silicate–based materials, which differs from the results of the present study. Ha [39] and Mustafa et al. [40] both demonstrated increased resin bond strength to BD with delayed restoration, attributing this to continued hydration and maturation of BD over time. However, important methodological differences may explain the discrepancy. In this study, specimens were subjected to a 14-day pH cycling regimen consisting of alternating demineralizing (pH 5.0) and remineralizing (pH 7.0) solutions to simulate a cariogenic oral environment, followed by thermocycling for 500 cycles between 5°C and 55°C. These aging protocols applied chemical and thermal stresses that more closely approximate intraoral conditions and may accelerate interfacial degradation. Prolonged maturation under such conditions may reduce the surface reactivity of BD, limit the availability of calcium ions for chemical interaction with functional adhesive monomers, and compromise micromechanical interlocking, thereby diminishing bond strength over time. In contrast, studies reporting improved bonding at longer intervals often employed storage in distilled water or artificial saliva without combined thermal and pH challenges, which may favor continued surface mineralization and apparent strengthening of the substrate [40].

Furthermore, Carretero et al. demonstrated that bond strength to BD is highly dependent on the adhesive strategy used [41]. Differences in adhesive systems and application protocols between previous studies and the present investigation may therefore further contribute to the observed variations in bonding outcomes. Previous studies have employed two main adhesive strategies with BD, ER and SE systems [18, 29–31, 33–35, 37, 38, 42, 43]. However, only a few studies have evaluated the SBS of universal adhesives used with BD [34–36]. Therefore, the second part of this study aimed to compare the bond strength of Single Bond Universal applied with ER or SE technique, as well as ER using Adper Single Bond 2. Our results showed that bond strength varied among the adhesive systems. Thus, the second null hypothesis was rejected. The specimens from the SU-ER group showed significantly higher bond strength than the other groups. This finding is supported by Cengiz and Ulusoy [29] and Meraji and Camilleri [30], who demonstrated that the use of the ER technique over BD enhanced the adhesion of RC. Only two studies have directly compared the same universal adhesive using both SE and ER techniques, as in our study [34, 36]. However, they found no statistically significant difference in SBS between SE and ER applications over BD. These differences between previous studies and the present findings may be attributed to variations in adhesive protocols and aging procedures. Moreover, our study demonstrated that AS-ER exhibited lower bond strength compared to SU-ER, which may be attributed to differences in the compositions of the two adhesives. Universal adhesive contains 10-MDP, a functional monomer reported to enhance bond strength through chemical interaction with calcium in calcium silicate-based materials, thereby complementing the existing micromechanical retention mechanisms [31, 34, 35, 37, 44, 45]. Additionally, the application of phosphoric acid prior to adhesive placement in the ER system may produce more distinct and retentive porosities on BD, facilitating deeper adhesive penetration compared to the SE approach [30, 35, 36]. The study by Anastasiadis et al. [46] supported this finding. They reported that treating the BD surface with phosphoric acid prior to adhesive application promoted both physical changes and alterations in the chemical composition of BD, through the formation of a calcium phosphate precipitate that enhances micromechanical interlocking. This may explain the significantly higher bond strength found for the universal adhesive system placed with the ER technique when compared to the SE system. While the present findings and some previous studies suggest that ER strategies may enhance adhesion, other authors have reported that prolonged phosphoric acid etching (exceeding 30 s) can adversely affect BD and reduce bond strength [47]. This indicates that the effect of acid etching on BD is highly dependent on etching time and protocol.

Regarding the failure mode in HVGIC groups, most of the failures were adhesive regardless of the maturation times. This result is in accordance with that of previous studies [29, 31]. The high incidence of adhesive failures between HVGIC and BD may indicate that the bond between the two materials is not strong. This is supported by earlier studies as well as our findings, where specimens exhibited pre-test failure due to GIC dislodging from the BD surface before SBS testing [29, 34]. Pre-test failure is associated with very low SBS between two materials. Various methods have been proposed to address this issue, including: (1) excluding all the pre-test failures and (2) including all the pre-test failures and recording them as either the lowest value measured in the group or as 0 MPa. In our study, pre-test failures were included in the analysis, with the SBS recorded as 0 MPa, in accordance with previous studies [16, 34, 48, 49]. Excluding all the pre-test failures from the analysis was not selected, as it could lead to an overestimation of the actual bond strength [16, 34]. In contrast to HVGIC, most failures in the RC groups occurred cohesively in the BD. The higher number of cohesive failures between BD and RC indicated lower compressive strength in BD compared to adhesive strength, and our result is similar to that of previous studies [29, 30, 36, 39]. It has been proposed that bonding between BD and RC is acceptable when fractures occur cohesively within BD [43].

In this study, pH cycling and thermocycling were applied to simulate the oral environment. It has been reported that a thermocycling regimen at a minimum of 500 cycles in water between 5°C and 55°C is an appropriate method for artificial aging [22]. However, the thermocycling process can adversely affect interfacial bonding and, consequently, the bond strength of calcium silicate cements to dentine. Meraji et al. reported a reduction in bond strength after thermocycling [30]. Furthermore, variations in the coefficients of thermal expansion between the bonded materials can generate mechanical stresses that further challenge the stability of the bonding interface [50, 51]. Therefore, the thermocycling process may have contributed to the low SBS observed in this study and may have even caused the pre-test failures in the HVGIC groups.

This study has some limitations. As an in vitro study, it does not fully replicate the biological, mechanical, and microbiological complexity of the oral environment, although oral simulation protocols, including pH cycling and thermocycling, were incorporated. These aging protocols may not entirely simulate long-term intraoral degradation or other clinical factors such as saliva, occlusal forces, and patient-related variables. Therefore, future in vivo studies and long-term clinical investigations are recommended to validate these findings and better clarify the clinical performance of restorations placed over BD.

## Conclusion

When bonded with BD, RC applied using a 10-MDP-containing universal adhesive in ER mode demonstrated higher bond strength than HVGIC, regardless of BD maturation time. Failure in the RC groups was predominantly cohesive, whereas HVGIC groups showed mainly adhesive failures. Immediate restoration using the 10-MDP-containing ER adhesive showed comparable performance to delayed placement, supporting its feasibility for single-visit procedures.

## Data Availability

The data used and/or analyzed during the current study are available from the corresponding author upon reasonable request.
